# Optimum contribution selection for conserved populations with historic migration

**DOI:** 10.1186/1297-9686-44-34

**Published:** 2012-11-15

**Authors:** Robin Wellmann, Sonja Hartwig, Jörn Bennewitz

**Affiliations:** 1 Institute of Animal Husbandry and Animal Breeding, University of Hohenheim, D-70599 Stuttgart, Germany

## Abstract

**Background:**

In recent decades, local varieties of domesticated animal species have been frequently crossed with economically superior breeds which has resulted in considerable genetic contributions from migrants. Optimum contribution selection by maximizing gene diversity while constraining breeding values of the offspring or *vice versa* could eventually lead to the extinction of local breeds with historic migration because maximization of gene diversity or breeding values would be achieved by maximization of migrant contributions. Therefore, other objective functions are needed for these breeds.

**Results:**

Different objective functions and side constraints were compared with respect to their ability to reduce migrant contributions, to increase the genome equivalents originating from native founders, and to conserve gene diversity. Additionally, a new method for monitoring the development of effective size for breeds with incomplete pedigree records was applied. Approaches were compared for Vorderwald cattle, Hinterwald cattle, and Limpurg cattle. Migrant contributions could be substantially decreased for these three breeds, but the potential to increase the native genome equivalents is limited.

**Conclusions:**

The most promising approach was constraining migrant contributions while maximizing the conditional probability that two alleles randomly chosen from the offspring population are not identical by descent, given that both descend from native founders.

## Background

Many local varieties of domesticated animal species have been established in the last centuries. However, due to agricultural innovations since the beginning of the 19th century and subsequent intensification of production, many landraces are no longer adapted to their changing environments [1,2]. They have been crossed with superior breeds in order to improve the economic value of the breeding stock. Gene flow usually occured from the economically most important breeds to the landraces, but not backwards. Consequently, most historic breeds are now extinct and the remaining ones have considerable genetic contributions from a small number of economically superior breeds. Efforts are needed to prevent the remaining historic breeds and their gene pools to become extinct. Conservation efforts can have different objectives. Objectives of breeding programs can be to breed back the historic breeds by removing genetic contributions of migrants, to conserve the breeds in their present appearance, or to increase the economic values. In any case, genetic contributions arising from more frequent breeds are not subject to conservation efforts since their genes are widespread.

Meuwissen [3] proposed to maximize the expected mean breeding value of the offspring while constraining its gene diversity to a predefined value. A related but not equivalent approach is to maximize the gene diversity in the offspring with or without constraining its expected mean breeding value to a predefined value. In this paper, the latter approach is applied and generalized. This approach seems more appropriate for conserved populations because for these populations the focus is on conservation. In general, the method consists of calculating an optimum contribution *c*_*a*_ (or the desired number of offspring) for each breeding individual *a* such that the offspring population maximizes an appropriate objective function *ϕ*under some side conditions.

In the classical approach [4] (Approach A) the gene diversity *GD* in the offspring *O*(**c**) is maximized, where the vector **c** contains the genetic contribution of each breeding individual to the offspring population. Thus, *ϕ*_*A*_(**c**) = *GD*(*O*(**c**)). Gene diversity of a population is the probability that two alleles randomly chosen from the population are not identical by descent (IBD). However, this objective function may be not appropriate for conserved populations because maximization of gene diversity could be achieved by maximization of genetic contributions of migrants. Thus, this approach could eventually lead to extinction of the native breeds. Gene diversity should not fall below a certain level in order to avoid inbreeding depression. Gene diversity is, however, not the parameter that should be maximized in conserved populations. In conserved populations, we are interested in the conservation of alleles that come from native founders, as migrant alleles usually originate from non-endangered breeds. That is, we want to maximize the probability *ϕ*_*B*_ that both alleles are not IBD and descended from native founders (Approach B), or the probability *ϕ*_*C*_ that both alleles are not IBD and at least one of them descended from native founders (Approach C). We also considered the possibility of maximizing the conditional probability *ϕ*_*D*_ that both alleles are not IBD, given that both descended from native founders (Approach D). For Approach D, we constrained the mean migrant contribution in the offspring population.

Lacy [5] introduced the concept of founder genome equivalents (FGE). The FGE of a population is the minimum number of unrelated founders that would be needed to establish a population that has the same gene diversity as the population under study. Recall that gene diversity is the probability that two alleles chosen at random are not IBD. However, a more important parameter to characterize the value of a breed for conservation purposes is the conditional probability that two randomly chosen alleles are not IBD, given that both descended from native founders. We call it the conditional gene diversity of the population. Large conditional gene diversity indicates that many native founder alleles have been retained in the population even though they may be at low frequencies. This has led to the following definition of the native genome equivalents (NGE) of a population as the minimum number of unrelated founders that would be needed to establish a population that has the same conditional gene diversity as the population under study. It can be interpreted as the FGE that originate from native founders and that are still present in the population. Besides maintaining the economic value of the breed, the main objective of a conservation program for a population with historic migration is to maximize the NGE and to minimize the genetic contributions of migrants simultaneously.

In this paper, we compare objective functions *ϕ*_*A*_,*ϕ*_*B*_, *ϕ*_*C*_ and *ϕ*_*D*_ with respect to their ability to conserve the gene diversity, to increase the FGE originating from native founders (i.e. the NGE), and to decrease the genetic contributions of migrants. Algorithms for solving these optimization problems are also derived and implemented in the R package *PedAnalysis*. Methods were applied and effective sizes were calculated for three German cattle breeds: Vorderwald, Hinterwald and Limpurg.

## Methods

### Definitions

Since the methods were applied to populations with overlapping generations, all definitions are based on birth cohorts rather than generations. A birth cohort *J* is a set of individuals born in a particular time interval, e.g. the individuals *B*_*t*_ born in year *t*, or the population *P*_*t*_ at time *t*[6]. Since the date of death is unknown in most cases, the population *P*_*t*_ consists of all individuals up to a particular age *T*. This age *T* could be the average age of individuals when their last offspring was born, or, for simplicity, it could be the generation interval *I*. Thus, population *P*_*t*_ consists of all individuals born in the time interval *t-T,t*.

The **gene diversity ***GD*(*J*) of birth cohort *J* is the probability that two alleles chosen at random from the birth cohort are not IBD. We can write

(1) $$\mathrm{GD}\left(J\right)=P(X_{J}\neq Y_{J}),$$

where alleles *X*_*J*_ and *Y*_*J*_ are randomly chosen with replacement from birth cohort *J*, and founder alleles are assumed to be pairwise different. An equivalent representation is $\mathrm{GD}(J)=1-{\bar{f}}_{J}$, where ${\bar{f}}_{J}$ is the average coancestry in birth cohort *J*.

Each allele descends from a particular founder. Take  to be the set of founder alleles. We distinguish between native founders and migrants, whereby a native founder is a founder that is not a migrant. A native founder is typically an individual with unknown pedigree that belongs to the population and was born before a certain date *t*_*s*_. A migrant is typically an individual that either comes from an other population (breed), or an individual with unknown pedigree that was born after the date. The date *t*_*s*_could be chosen shortly after establishment of the stud book when a sufficient portion of the population was recorded. We can write

(2) A=F∪ℳ,

where  is the set of alleles that come from native founders and > is the set of alleles that come from migrants.

We define the **conditional gene diversity***condGD*(*J*) of birth cohort *J* as the conditional probability that two alleles randomly chosen from the birth cohort are not IBD, given that both descend from native founders. That is,

(3) $$\mathrm{condGD}(J)=P(X_{J}\neq Y_{J}|X_{J}\in F,Y_{J}\in F).$$

The **founder genome equivalents***FGE*(*J*) of birth cohort *J* is defined as the minimum number of founders that would be needed to establish a population that has the same gene diversity as the individuals in birth cohort *J*. It can be computed as

(4) $$\mathrm{FGE}(J)=\frac{1}{2{\bar{f}}_{J}}=\frac{1}{2(1-\mathrm{GD}(J))},$$

see [4]. Analogously, we define the **native genome equivalents***NGE*(*J*) of birth cohort *J* as the minimum number of founders that would be needed to create a population that has the same conditional gene diversity as the individuals in birth cohort *J*. We have

(5) $$\mathrm{NGE}(J)=\frac{1}{2(1-\mathrm{condGD}(J))}.$$

However, a problem with this definition is that native founders of the population are assumed to be unrelated, which is not true. As a consequence, in the first generation the NGE would be almost as large as the total population size. However, due to the invalid assumption of unrelated founders, the limited effective size causes the NGE to decrease tremendously shortly after the last native founders have entered the population. In order to avoid this artifact, we extrapolate the history of the breed back in time and use as the reference population not the founders listed in the stud book, but the population at an earlier time *t*_0_. That is, all individuals are assumed to be unrelated in year *t*_0_. In the applications, the base year was *t*_0_ = 1800. We define the conditional gene diversity of an age cohort *J*_*t*_at time *t* ≥ *t*_*s*_ with respect to base year *t*_0_ as

(6) $$\mathrm{condG}D_{t_{0}}(J_{t})={\left(1-\frac{1}{2\mathrm{hist}N_{e}}\right)}^{\frac{t_{s}-t_{0}}{I}}\frac{\mathrm{condGD}(J_{t})}{\mathrm{condGD}(P_{t_{s}})},$$

where $P_{t_{s}}$ is the population at time *t*_*s*_, *I* is the generation interval, and *histN*_*e*_ is the historic effective size of the population. The historic effective size can be estimated from marker data [7]. The term that defines the conditional gene diversity is the product of two factors. The first is the estimated gene diversity in the population at time *t*_*s*_, and the second is the factor by which the conditional gene diversity decreased between *t*_*s*_ and *t*. Consequently, the **NGE with respect to base year***t*_0_ can be calculated as

(7) $$\mathrm{NG}E_{t_{0}}(J_{t})=\frac{1}{2(1-\mathrm{condG}D_{t_{0}}(J_{t}))}.$$

A further parameter that can be of interest is the **effective size** of the population. The effective size *N*_*e*_(*t*_1_*t*_2_) of a population within a time interval *t*_1_*t*_2_ is the size of an idealized random mating population of constant size that causes the same decrease of gene diversity as the true population within $\frac{t_{2}-t_{1}}{I}$ generations. However, in breeds with steady gene flow from other populations, the gene diversity does not decrease below a certain level, so this definition of the effective size does not make much sense for populations with migration. Therefore, we use a slightly different definition. We define the **native effective size***N*_*eN*_(*t*_1_*t*_2_) as the size of an idealized random mating population of constant size that causes the same decrease of the *conditional* gene diversity *condGD*(*P*_*t*_) as the true population within $\frac{t_{2}-t_{1}}{I}$ generations. The effective population size at time *t*, defined as *N*_*eN*_(*t*) = lim_*ε*→0_*N*_*eN*_([*t* − *ε*, *t* + *ε*]), was calculated as described in [8], except that it was calculated from the conditional gene diversity. The native effective size quantifies the decrease of genome equivalents originating from native founders because the NGE depend only on the conditional gene diversity, as can be seen from the previous two equations. In a population without migration, *N*_*e*_ and *N*_*eN*_are equal. However, in a population with steady gene flow from other populations, *N*_*eN*_ is smaller than *N*_*e*_ because the gene diversity approaches a plateau level, so *N*_*e*_(*t*) goes to infinity.

The population *P*_*t*_at time *t*, which consists of all individuals up to an age of *T* years, has gene diversity *GD*(*P*_*t*_), native genome equivalents $\mathrm{NG}E_{t_{0}}(P_{t})$, and genetic contribution $C_{F}(P_{t})$ from native founders. Note that $C_{F}(J)=P(X_{J}\in F)$, so $C_{F}(J)$ is the probability that a randomly chosen allele from age cohort *J* descends from a native founder. Besides monitoring of these quantities, a major task for a conservation program is the calculation of optimal genetic contributions for the breeding individuals that maximize the conditional gene diversity in the offspring and simultaneously maximize the genetic contribution from native founders in the offspring. Moreover, a sufficient level of gene diversity must be maintained in order to avoid inbreeding depression. In general, however, the quantities $\mathrm{NG}E_{t_{0}}(J)$ and $C_{F}(J)$ cannot be maximized simultaneously, so an objective function is needed that considers each appropriately.

The usual approach (Approach A) for populations without migration is the calculation of genetic contributions $c_{t}^{A}$ for the breeding individuals of population *P*_*t*_such that the gene diversity

(8) $$\phi_{A}(J)=\mathrm{GD}(J)=P(X_{J}\neq Y_{J})$$

is maximized by a hypothetical (infinitely large) offspring population $O_{t}(c_{t}^{A})$. This approach is called minimum kinship selection [9]. Note that the gene diversity $\mathrm{GD}(O_{t}(c_{t}^{A}))=\phi_{A}(O_{t}(c_{t}^{A}))$ of the hypothetical offspring is known as the potential diversity of the population at time *t*[6]. A more appealing approach for populations with migration is to use genetic contributions $c_{t}^{B}$ for the breeding individuals such that the probability

(9) $$\phi_{B}(J)=P(X_{J}\neq Y_{J}\;\;\mathrm{and}\;\;X_{J}\in F\;\;\mathrm{and}\;\;Y_{J}\in F)$$

is maximized by the resulting offspring population $O_{t}(c_{t}^{B})$. This is the probability that two randomly chosen alleles from the offspring are not IBD and are from native founders (Approach B). As a third approach, we consider maximization of the probability that two randomly chosen alleles from the offspring are not IBD and at least one of them descends from a native founder (Approach C). In this case, genetic contributions $c_{t}^{C}$ for the breeding individuals are calculated such that the offspring population $O_{t}(c_{t}^{C})$ maximizes

(10) $$\phi_{C}(J)=P(X_{J}\neq Y_{J}\;\;\mathrm{and}\;\;(X_{J}\in F\;\;\mathrm{or}\;\;Y_{J}\in F)).$$

Finally, we consider maximizing the conditional gene diversity in the offspring population. That is, genetic contributions $c_{t}^{D}$ for the breeding individuals were calculated such that the conditional probability

(11) $$\phi_{D}(J)=P(X_{J}\neq Y_{J}\;|\;X_{J}\in F\;\;\mathrm{and}\;\;Y_{J}\in F)$$

is maximized. This approach is intuitively appealing because it maximizes NGE. It has, however, the disadvantage that the conditional gene diversity can be large even for offspring populations with very large migrant contributions. This is due to conditioning on the event that the randomly chosen alleles *X*_*J*_ and *Y*_*J*_originate from native founders. This can be seen as follows. Take a solution $c_{t}^{D}$ of the optimization problem and suppose that at least one migrant is a potential breeding individual. Then it can be shown mathematically that the genetic contribution of this migrant to the offspring population can be arbitrarily increased without changing the value of the objective function. Thus, the solution of the optimization problem may be not unique, and one solution maximizes migrant contributions. In order to avoid this, we put an additional constraint on the maximum permissible value for the genetic contribution from migrants to the offspring population.

### Computations

To calculate the parameters defined in the previous section, the following quantities are needed. First, the coancestry *f*_*i*,*j*_ is needed for each pair of individuals *i,j*. It is the probability that two alleles randomly chosen from the individuals are IBD. That is,

(12) $$f_{i,j}=P(X_{i}=X_{j}),$$

where allele *X*_*i*_ is randomly chosen from the two alleles of individual *i* at a particular locus.

Now we define an equivalence relation on the set of founder alleles. Two alleles *x*_*i*_,*x*_*j*_ are equivalent (*x*_*i*_≡_*M*_*x*_*j*_) if they are IBD or if both are migrant alleles. For two alleles randomly chosen from individuals *i, j*, the probability for this to occur is

(13) $$\begin{array}{lll} f_{i,j}^{M} & =P(X_{i}\equiv_{M}X_{j})\; & \;\\ & =P\left(X_{i}=X_{j}\;\;\mathrm{or}\;\;(X_{i}\in ℳ,X_{j}\in ℳ)\right).\; & \; \end{array}$$

A second equivalence relation is defined as follows. Two alleles *x*_*i*_,*x*_*j*_ are equivalent (*x*_*i*_≡_*FM*_*x*_*j*_) if both are native founder alleles or if both are migrant alleles. For two alleles randomly chosen from individuals *i, j*, the probability for this to occur is

(14) $$\begin{array}{lll} f_{i,j}^{\mathrm{FM}} & =P(X_{i}\equiv_{\mathrm{FM}}X_{j})\; & \;\\ & =P\left(\left(X_{i}\in F,X_{j}\in F)\;\;\mathrm{or}\;\;(X_{i}\in ℳ,X_{j}\in ℳ\right)\right).\; & \; \end{array}$$

These probabilities have the advantage that they can easily be computed with existing software, e.g. with function *kinship()* from the R-package *kinship*. For calculation of $f_{i,j}^{M}$, the parents of all migrants were identified with the same dummy individual and for this individual a pedigree with several generations of selfing was added. The coancestry of individuals *i, j*, computed from this extended pedigree is equal to $f_{i,j}^{M}$. Equality holds only approximately because only a finite number of generations of selfing was added. For calculation of $f_{i,j}^{\mathrm{FM}}$, the parents of all migrants were identified with one single dummy individual, the parents of all native founders were identified with another single dummy individual, and for both individuals pedigrees with several generations of selfing were added. The coancestry of individuals *i, j*, computed from this extended pedigree, is equal to $f_{i,j}^{\mathrm{FM}}$. For example, consider two full sibs *i, j* whose sire is a migrant and whose dam is a native founder. Their coancestry is $f_{\mathrm{ij}}=\frac{1}{4}$, but $f_{\mathrm{ij}}^{M}=\frac{3}{8}$, and $f_{\mathrm{ij}}^{\mathrm{FM}}=\frac{1}{2}$.

Let $f_{P_{t}}$ be the *N*_*t*_×*N*_*t*_ coancestry submatrix for the *N*_*t*_ individuals from population *P*_*t*_ that is obtained from the true pedigree (i.e., $f_{P_{t}}={\left(f_{\mathrm{ij}}\right)}_{i,j\in P_{t}})$. The *N*_*t*_×*N*_*t*_ matrix that contains the probabilities $f_{i,j}^{M}$ for each pair of individuals *i, j* from population *P*_*t*_is denoted as $f_{P_{t}}^{M}={\left(f_{\mathrm{ij}}^{M}\right)}_{i,j\in P_{t}}$, and the *N*_*t*_×*N*_*t*_ matrix that contains the probabilities $f_{i,j}^{\mathrm{FM}}$ is denoted as $f_{P_{t}}^{\mathrm{FM}}={\left(f_{\mathrm{ij}}^{\mathrm{FM}}\right)}_{i,j\in P_{t}}$. That is, rows and columns that correspond to individuals not born in time interval [*t-T,t*] and dummy individuals were excluded from the matrix.

Additionally, the *N*_*t*_-dimensional vector $C_{t}={\left(C_{t1},\ldots ,C_{tN_{t}}\right)}^{T}$ is needed and contains the genetic contribution of native founders for each individual of population *P*_*t*_. Note that $C_{F}(P_{t})$ is the mean of vector **C**_*t*_. Let ${\bar{f}}_{P_{t}},{\bar{f}}_{P_{t}}^{M}$, and ${\bar{f}}_{P_{t}}^{M}$ be the means of the respective matrices. It is well known that the gene diversity can be computed as [4]

(15) $$\mathrm{GD}(P_{t})=1-{\bar{f}}_{P_{t}}.$$

Proofs of all numbered equations are presented in Additional file 1, in which it is shown that the conditional gene diversity satisfies

(16) $$\mathrm{condGD}(P_{t})=\frac{{\bar{f}}_{P_{t}}^{\mathrm{FM}}-{\bar{f}}_{P_{t}}^{M}}{C_{F}\left(P_{t}\right)-\frac{1-{\bar{f}}_{P_{t}}^{\mathrm{FM}}}{2}}.$$

Let $O_{t}^{N}(c)$ be an arbitrary (hypothetical) offspring population of size *N* that is obtained from population *P*_*t*_ such that each breeding individual *a* ∈ *P*_*t*_ has genetic contribution *c*_*a*_to the offspring population. The probability that an allele randomly chosen from the offspring population descends from a native founder is

(17) $$C_{F}(O_{t}(c))=C_{F}(O_{t}^{N}(c))=c^{T}C_{t},$$

and the conditional gene diversity in the offspring population is

(18) $$\begin{array}{lll} \mathrm{condGD}(O_{t}(c)) & =\underset{N\to \infty }{\lim}\mathrm{condGD}(O_{t}^{N}(c))\; & \;\\ & =\frac{c^{T}(f{\;}_{P_{t}}^{\mathrm{FM}}-f_{P_{t}}^{M})c}{c^{T}C_{t}-\frac{1-c^{T}f{\;}_{P_{t}}^{\mathrm{FM}}c}{2}}.\; \end{array}$$

It is well known that

(19) $$\underset{N\to \infty }{\lim}\phi_{A}(O_{t}^{N}(c))=1-c^{T}f_{P_{t}}c,$$

so the optimum contributions $c_{t}^{A}$ for the breeding individuals with respect to objective function *ϕ*_*A*_minimize $c^{T}f_{P_{t}}c$ under side conditions *c*_*a*_ ≥ 0 and $\underset{a}{\sum }c_{a}=1$. Additional side conditions can be added to fulfil biological and practical requirements. Moreover, we have

(20) $$\underset{N\to \infty }{\lim}\phi_{B}(O_{t}^{N}(c))=c^{T}(f{\;}_{P_{t}}^{\mathrm{FM}}-f{\;}_{P_{t}}^{M})c,$$

so the optimum contributions $c_{t}^{B}$ for the breeding individuals with respect to objective function *ϕ*_*B*_ minimize $c^{T}(11^{T}-(f{\;}_{P_{t}}^{\mathrm{FM}}-f{\;}_{P_{t}}^{M}))c$ under the side conditions described above, where **1** is a vector with ones. Since

(21) $$\underset{N\to \infty }{\lim}\phi_{C}(O_{t}^{N}(c))=1-c^{T}f{\;}_{P_{t}}^{M}c,$$

the optimum contributions $c_{t}^{C}$ for the breeding individuals with respect to objective function *ϕ*_*C*_ minimize $c^{T}f{\;}_{P_{t}}^{M}c$ under the side conditions. Finally, we have

(22) $$\underset{N\to \infty }{\lim}\phi_{D}(O_{t}^{N}(c))=\frac{c^{T}(f{\;}_{P_{t}}^{\mathrm{FM}}-f{\;}_{P_{t}}^{M})c}{c^{T}Q_{t}c},$$

where $Q_{t}=\frac{1}{2}\left(C_{t}1^{T}+1C_{t}^{T}-11^{T}+f_{P_{t}}^{\mathrm{FM}}\right)$ is a *N*_*t*_ × *N*_*t*_ matrix. This function was maximized under the side conditions described above. Moreover, the additional side constraint $c^{T}C_{t}\geq c_{F}$ was applied, where $c_{F}$ is the minimum permissible contribution of native founders to the offspring population. This is a quadratic fractional programming problem with linear constraints, so the objective function could have multiple local maxima. As mentioned in the previous section, one solution of the optimization problem maximizes migrant contributions, so the inequality constraint could be replaced by the equality constraint $c^{T}C_{t}=c_{F}$. For each offspring population *J* that satisfies this equality constraint, the objective function (i.e. the conditional gene diversity) satisfies

(23) $$\phi_{D}\left(J\right)\approx \frac{\phi_{B}(J)}{P{\left(X_{J}\in F\right)}^{2}}=\frac{\phi_{B}(J)}{c_{F}^{2}}\propto \phi_{B}\left(J\right),$$

where the approximation is exact if the events $X_{J}\in F$ and $Y_{J}\in F$ are independent. Therefore, an approximate solution was obtained by maximizing objective function *ϕ*_*B*_ under the additional constraint $c^{T}C_{t}=c_{F}$. The resulting contributions for the breeding individuals were used as starting values for general nonlinear optimization in order to obtain the exact solution. In the applications, the threshold value $c_{F}$ was quite arbitrarily chosen as the 75% quantile of the genetic contributions from native founders to individuals in the population. The same quantile was used for all breeds and years in order to make the results comparable. Results could be improved by choosing breed dependent threshold values.

We used the interior point method *ipop* in R-package *kernlab* (see [10]) for objective functions *ϕ*_*B*_ and *ϕ*_*D*_, whereas for objective functions *ϕ*_*A*_and *ϕ*_*C*_with positive definite matrices we used *solve.QP* from R-package *quadprog*. It implements the dual method of Goldfarb and Idnani [11,12].

### Materials

Only three local cattle varieties of Baden and Württemberg in the south-west of Germany have been preserved from extinction. These are the Vorderwald cattle, Hinterwald cattle, and Limpurg cattle. Other local breeds were replaced by Simmentaler Fleckvieh after their introduction at the beginning of the 19th century because the small landraces were not suitable for tillage [1].

The small *Hinterwald cattle* could be preserved as an almost pure breed until the beginning of the 20th century [13,14] because the poor soil quality in its region of origin was not suitable for larger breeds. Nevertheless, this breed adopted the colour of the Simmentaler Fleckvieh during the 19th century [15]. The Hinterwald cattle were occasionally crossed with the Vorderwald cattle [16] and with Fleckvieh.

The red-and-white marked, colour-sided [17]*Vorderwald cattle* were frequently crossed with Simmentaler cattle. Consequently, the white stripe along the back became rare already around 1900 [16]. After the Second World War, Vorderwald cattle were also crossed with Ayrshire, Red Holstein and Montbéliard cattle in order to improve milk yield. These crosses were registered as Vorderwald cattle. Extinction probabilities for Vorderwald and Hinterwald cattle were estimated by [18].

The yellow coloured *Limpurg cattle* were not only frequently crossed with Simmentaler cattle [19], but also occasionally with Braunvieh and Gelbvieh cattle [15] in order to increase body size. Nevertheless, the population size decreased dramatically. Only 17 Limpurg cows were registered in 1967, so the breeding association was dissolved. Several Limpurg cattle, however, were rediscovered in 1986 and a new stud book was established. Not only Limpurg cattle were registered, but also Fleckvieh crosses, and some Gelbvieh and Glan-Donnersberger bulls [16].

The data consisted of the pedigrees and additional information on 25 412 Hinterwald cattle, 185 315 Vorderwald cattle, and 4 150 Limpurg cattle. Vorderwald cattle without offspring were removed from the data in order to reduce the data set. Pedigrees of Hinterwald and Vorderwald cattle trace back only to 1948 because the stud books were renewed after the Second World War. Pedigrees of Limpurg cattle trace back only to 1970. Cattle from other breeds were considered to be migrants. Additionally, Hinterwald and Vorderwald cattle with unknown pedigree born after *t*_*s*_ = 1970 were also considered migrants, although some may have purebred ancestors. Limpurg cattle with unknown pedigree were considered to be migrants if they were born after *t*_*s*_ = 1988. The generation intervals were similar for the three breeds (unpublished results). Here, we assumed a generation interval of *I* = 5.3 years for all breeds.

## Results

**Figure 1:**
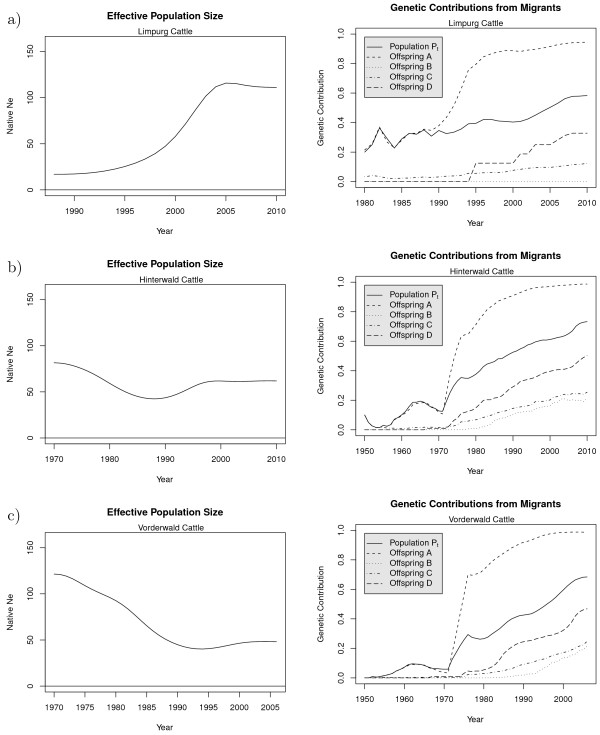
**Native effective size and migrant contributions.** Native effective size *N*_*eN*_(left) and genetic contributions from migrants (right) in the real population *P*_*t*_and in the hypothetical offspring populations for selection strategies A, B, C, and D (right) for **(a)**: Limpurg cattle, **(b)**: Hinterwald cattle, **(c)**: Vorderwald cattle.

The left hand side of Figure 1 shows the development of the native effective size *N*_*eN*_ for the three breeds. Around 1990, the effective size of Limpurg cattle was only about 20, which was due to the small population size. However in most cases, the effective size was above 50 for all three breeds. In 2011, 7952 Vorderwald cows, 2328 Hinterwald cows, and 471 Limpurg cows were registered. Interestingly, there appeared to be no relationship between the effective size and the total population size when the number of individuals exceeds the minimum number required to reach an *N*_*e*_of approximately 50.

The right hand side of Figure 1 shows for each breed how the genetic contributions of migrants changed over time. Migrant contributions are shown for the true population *P*_*t*_ and for the hypothetical offspring populations that would be obtained if optimum contribution selection were applied to population *P*_*t*_. The solid lines show that migrant contributions increased steadily for all three breeds. The dashed line for offspring A shows that all three breeds would become extinct if optimum contribution selection were used to maximize the gene diversity in the offspring. In contrast, objective functions *ϕ*_*B*_ and *ϕ*_*C*_ would reduce migrant contributions substantially by more than 50% in all three breeds. According to the constraint applied for objective function *ϕ*_*D*_, the corresponding line shows the 25% quantile of the migrant contributions in the population.

**Figure 2:**
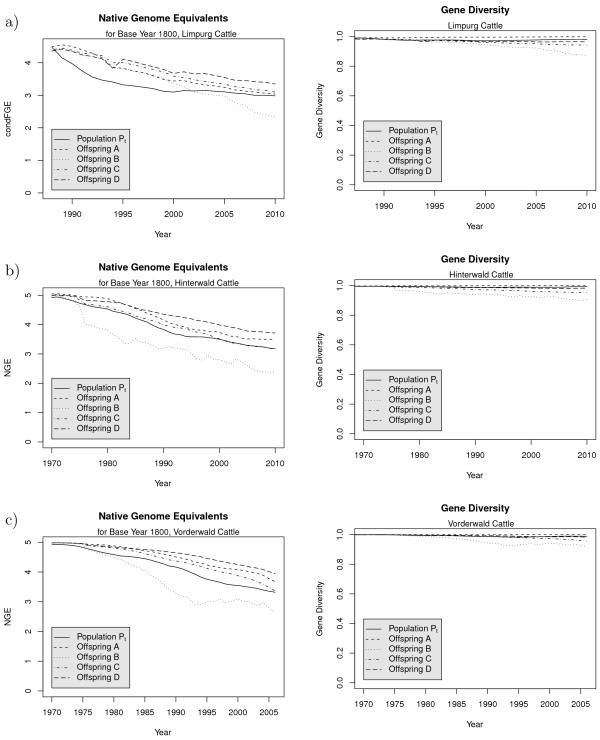
**Native genome equivalents and gene diversities.** Genome equivalents originating from native founders NGE (left) and gene diversity (right) in the real population *P*_*t*_and in the hypothetical offspring populations for selection strategies A, B, C, and D for **(a)**: Limpurg cattle, **(b)**: Hinterwald cattle, **(c)**: Vorderwald cattle.

The left hand side of Figure 2 shows the development of NGE for the true population and for the hypothetical offspring populations. We used the year *t*_0_ = 1800 as the base year. The historic *N*_*e*_ is not known for these breeds. In the figure, we assumed a historic *N*_*e*_of 150 for each breed, which is in good accordance with the results obtained by [20] for various cattle breeds during this period of time. For the population in 2005, the computed NGE with respect to base year *t*_0_ was 3.1 for Limpurg cattle, 3.3 for Hinterwald cattle, and 3.2 for Vorderwald cattle. For comparison, the NGE computed under the assumption of unrelated founders was 7.3 for Limpurg cattle, 8.3 for Hinterwald cattle, and 8.0 for Vorderwald cattle. Figure 2 shows that the NGE would decrease by using objective function *ϕ*_*B*_. This suggests that the individuals with the smallest migrant contributions are closely related, so they share the same founder alleles. The use of objective function *ϕ*_*C*_would cause a small increase of the NGE for all three breeds. If the constraint on migrant contributions is not too serious, then objective function *ϕ*_*D*_would cause the largest increase in NGE. However, the potential to increase NGE is limited.

The right hand side of Figure 2 shows the changes in gene diversity. It can be seen that the gene diversity is high for all three breeds. This is caused by migration. Note that the native effective population size quantifies the decrease of genome equivalents arising from native founders, so the gene diversity can be constant (or increase due to migration) even if the native effective population size is small. As expected, optimum contribution selection with objective functions *ϕ*_*B*_, *ϕ*_*C*_, or *ϕ*_*D*_ would cause a moderate but an acceptable loss of gene diversity.

## Discussion

Most of the time, the native effective size *N*_*eN*_was above 50 for the three breeds and due to migration, *N*_*e*_ was larger than *N*_*eN*_. An effective size of at least 50 is considered acceptable, although an *N*_*e*_of 100 is recommended to be on the safe side [21]. Many cattle breeds have effective sizes between 50 and 100 regardless of the total population size. Therefore, in order to conserve the overall gene diversity, it is generally recommended to conserve a large number of breeds with small population sizes rather than a small number of breeds with large population sizes. In this case, different alleles would be preserved in different subpopulations. These populations can be used as resources to identify advantageous genes that can be introgressed into commercial populations. Conserved populations must be sufficiently large to allow for this. However, breeds that are close to the economic viability threshold and populations that are expected to occupy niches that are different from that of established commercial breeds, should have larger population sizes in order to enable a sufficient selection response. Examples of the importance of farm animal genetic resources are the introgression of the polled gene into economically important cattle breeds, the introduction of indicine cattle breeds to South America because of their adaption to extreme environments, and introgression of genes for disease resistance into highly productive susceptible breeds [22].

The current *N*_*eN*_of the Vorderwald cattle was smaller than the estimates of the effective size obtained by [23] with other methods. The reason is probably that other methods do not distinguish between migrants and native founders. Genome equivalents arising from native founders are likely to decline faster than those arising from migrants because migrants are usually from economically superior breeds. The sufficiently large *N*_*eN*_ show that for all three breeds, migration from other breeds was much larger than it was needed to avoid unacceptably high inbreeding depression. As a consequence, these breeds share only a small portion of their genes with the corresponding historic breeds of the same name. We showed that it is still possible to substantially increase the genetic contribution from the historic breeds by optimum contribution selection.

For optimum contribution selection, the choice of the objective function was crucial. Maximization of gene diversity (Approach A) turned out to substantially increase the migrant contributions and thus would lead to the extinction of these breeds. Approach B has the desired effect to substantially decrease the migrant contributions but does not put enough weight on the conservation of gene diversity. It is not recommended because it would reduce NGE and cause the largest loss of gene diversity. Approach C is recommended for conserved populations because for all three breeds the use of this objective function substantially decreased the migrant contributions, increased the NGE, and caused only a moderate decrease of gene diversity. Approach D can also be recommended, although it requires choice of a threshold for the migrant contributions. If the threshold is chosen appropriately, then this approach causes the largest increase in NGE. However, the potential to increase the NGE was small for the breeds considered. Interestingly, for the current populations, optimum contributions for Approach A were slightly negatively correlated with the optimum contributions obtained for the other approaches, whereas the optimum contributions for the approaches B, C, and D were pairwise positively correlated (not shown).

Amador *et al.*[24] proposed two other approaches to reduce migrant contributions. Their first approach was to minimize migrant contributions in the offspring population. Their second approach was to minimize the probability that two alleles randomly chosen from the offspring population are IBD and descend from migrants. This objective function was computed from partial coancestry coefficients [25], but could also be computed by the methodology introduced in this paper. For both approaches, the maximum rate of inbreeding was restricted. However, provided that an acceptable rate of inbreeding can be achieved, it is not obvious why it is desirable that alleles originating from migrants should be not IBD in the offspring population. In contrast, all approaches proposed in this paper aim at increasing the probability that alleles originating from native founders are not IBD. Amador *et al.*[24] concluded that even with only a few generations without management, a small amount of introgression can spread into the population and it may be almost impossible to recover. This was not observed in our study. The reason is probably that the total population sizes of the cattle breeds were much larger than their effective sizes, which increased the probability to find individuals with small migrant contributions. Moreover, the cattle populations may deviate from random mating populations because some breeders avoid the use of bulls with high migrant contributions.

Another approach could be to minimize the effective number of non-founders *N*_*enf*_, as defined by Caballero and Toro [4], in the offspring population. This approach would be equivalent to maximization of ${\bar{f}}_{O}-\frac{1}{2N_{\mathrm{ef}}(O)}$ which would be achieved by increasing the average relationship ${\bar{f}}_{O}$ in the offspring population *O* and by increasing the effective number *N*_*ef*_ of founders in the offspring generation. Thus, the rate of inbreeding would have to be restricted by this alternative approach. This approach, however, would by definition not be optimal with respect to the objective functions introduced in this paper.

Our results show that migrant contributions can be substantially decreased for all three breeds, but the potential to increase the NGE is limited. The reduction of migrant contributions would be largely achieved in the first generation of management. In subsequent generations, some further improvement would be possible due to biological restrictions in previous generations. However, thereafter the management method becomes equivalent to an equalization of family sizes and no further reduction of migrant contributions could be achieved. Moreover, pedigree-based optimum contribution selection cannot remove genetic contributions of migrants that arose before recording of pedigrees started. However, removal of migrant contributions that arose earlier can be done subsequent to pedigree-based optimum contribution selection by identification of chromosome segments that are also present in the migrant breeds and by removal of those individuals with large migrant contributions from the breeding pool. Since migrants are usually males, haplotype variants of the Y-chromosome can be used as markers for paternal lineage [26] to identify the migrant breeds. For individuals that are not removed from the breeding pool (i.e individuals with small migrant contributions), optimum contributions can be calculated based on genomic relationships. In order to avoid that this approach causes the frequencies of migrant alleles to increase, the set of breeding individuals could be enlarged with individuals of the migrant breeds. After the optimum contributions have been computed, the contributions of these additional migrant individuals are set to zero, and the optimum contributions for individuals of the breed of interest are rescaled, so that they add up to one. Thereafter, it would be beneficial to combine closely related breeds with low gene diversity in order to reduce extinction probabilities [27], and to split breeds with a high gene diversity into several subpopulations in order to reduce the decrease of overall gene diversity [28]. Breeds with highest value for conservation should be given priority [29]. These breeds are likely found near the domestication center (since genetic diversity declines with increasing distance from the domestication centre [30]), far from the native areas of economically superior breeds, or live in harsh environmental conditions. Candidates are also breeds that are used for uncommon purposes (e.g. fighting cattle, cattle breeds used for cow racing).

## Conclusions

The usual recommendation to optimize contributions for breeding individuals by maximizing gene diversity in the offspring is not suitable for populations with historic migration because maximization of gene diversity would be achieved by maximization of migrant contributions. Thus, this approach, applied to populations with migration, would rapidely lead to their extinction. Two approaches can be recommended. The first is to maximize the probability that two alleles randomly chosen from the offspring population are not IBD and that at least one of them descended from a native founder (Approach C). The other approach is to constrain migrant contributions while maximizing the conditional probability that two alleles randomly chosen from the offspring population are not IBD, given that both descended from native founders (Approach D). Migrant contributions could be substantially decreased for the three breeds investigated here, but the potential to increase the NGE is limited.

Programs for pedigree-based optimum contribution selection and for the analyses presented in this paper are available in R package *PedAnalysis* from the first author. Since migrants are usually from genetically superior breeds, optimum contribution selection is likely to reduce breeding values if there is no constraint on the expected breeding value of the offspring. The program for optimum contribution selection allows adding the constraint that the expected mean breeding value of the offspring does not fall below a certain value. Moreover it is possible to put a constraint on the maximum number of offspring per male and female.

## Competing interests

The authors declare that they have no competing interests.

## Authors’ contributions

RW developed and implemented the methods and drafted the manuscript. SH prepared the data files and applied the methods. JB conceived the project and helped in drafting the manuscript. All authors have read and approved the final manuscript.

## Supplementary Material

Additional file 1 Proofs. The file provides the proofs of all numbered equations. Click here for file
