# Supplementary material for: Optimum contribution selection for conserved populations with historic migration
Source: Genet Sel Evol. 2012 Nov 15;44(1):34. doi: 10.1186/1297-9686-44-34 (PMC3807754; doi:10.1186/1297-9686-44-34)
Supplement: Additional file 1 — Proofs. The file provides the proofs of all numbered equations. [file 1297-9686-44-34-S1.pdf]

In the computations section, the matrices $f_{P_t}, f^M_{P_t}$, and $f^{FM}_{P_t}$ were defined for population $P_t$. More generally, let $f_J, f^M_J$, and $f^{FM}_J$ be the respective matrices for an arbitrary birth cohort $J$. Take $X_{1i}$ to be the paternal and $X_{2i}$ the maternal allele of individual $i$, and let $X_i$ be an allele at the locus that is randomly chosen from the two alleles of individual $i$. Let $s_i$ be the sire and $d_i$ the dam of individual $i$.

**Equation**

$$condGD(P_t) \;=\; \frac{\overline{f}^{FM}_{P_t} - \overline{f}^{M}_{P_t}}{C_{\mathcal{F}}(P_t) - \frac{1-\overline{f}^{FM}_{P_t}}{2}}. \tag{1}$$

**Proof:**

For individuals $i, j$ we have

$$\begin{aligned}
1 - f^{FM}_{ij} &= P(X_i \not\equiv_{FM} X_j) \\
&= P((X_i \in \mathcal{M} \text{ or } X_j \in \mathcal{M}) \text{ and } (X_i \in \mathcal{F} \text{ or } X_j \in \mathcal{F})) \\
&= P(X_i \in \mathcal{F},\ X_j \in \mathcal{M}) + P(X_i \in \mathcal{M},\ X_j \in \mathcal{F})
\end{aligned}$$

and

$$\begin{aligned}
1 - f^{M}_{ij} &= P(X_i \not\equiv_{M} X_j) \\
&= P(X_i \neq X_j \text{ and } (X_i \in \mathcal{F} \text{ or } X_j \in \mathcal{F})) \\
&= P(X_i \neq X_j \text{ and } (X_i \in \mathcal{F} \text{ and } X_j \in \mathcal{F})) \\
&+ P(X_i \neq X_j \text{ and } (X_i \in \mathcal{F} \text{ and } X_j \in \mathcal{M})) \\
&+ P(X_i \neq X_j \text{ and } (X_i \in \mathcal{M} \text{ and } X_j \in \mathcal{F})) \\
&= P(X_i \neq X_j \text{ and } X_i \in \mathcal{F} \text{ and } X_j \in \mathcal{F}) \\
&+ P(X_i \in \mathcal{F},\ X_j \in \mathcal{M}) + P(X_i \in \mathcal{M},\ X_j \in \mathcal{F}).
\end{aligned}$$

Thus,

$$\begin{aligned}
1 - \overline{f}^{FM}_{J} &= P(X_J \in \mathcal{F},\ Y_J \in \mathcal{M}) + P(X_J \in \mathcal{M},\ Y_J \in \mathcal{F}) \\
&= 2P(X_J \in \mathcal{F},\ Y_J \in \mathcal{M})
\end{aligned} \tag{7}$$

and

$$\begin{aligned}
1 - \overline{f}^{M}_{J} &= P(X_J \in \mathcal{F},\ Y_J \in \mathcal{M}) + P(X_J \in \mathcal{M},\ Y_J \in \mathcal{F}) \\
&+ P(X_J \neq Y_J \text{ and } X_J \in \mathcal{F} \text{ and } Y_J \in \mathcal{F}) \\
&= 2P(X_J \in \mathcal{F},\ Y_J \in \mathcal{M}) + P(X_J \neq Y_J \text{ and } X_J \in \mathcal{F} \text{ and } Y_J \in \mathcal{F}) \\
&= 1 - \overline{f}^{FM}_{J} + P(X_J \neq Y_J \text{ and } X_J \in \mathcal{F} \text{ and } Y_J \in \mathcal{F}).
\end{aligned}$$

It follows that

$$
\begin{aligned}
P(X_J \in \mathcal{F}, Y_J \in \mathcal{F}) \cdot condGD(J) &= P(X_J \neq Y_J \text{ and } X_J \in \mathcal{F} \text{ and } Y_J \in \mathcal{F}) \quad (8)\\
&= 1 - \overline{f}_J^{M} - (1 - \overline{f}_J^{FM})\\
&= \overline{f}_J^{FM} - \overline{f}_J^{M}
\end{aligned}
$$

and

$$
\begin{aligned}
P(X_J \in \mathcal{F},\, Y_J \in \mathcal{F}) &= P(X_J \in \mathcal{F}) - P(X_J \in \mathcal{F},\, Y_J \in \mathcal{M})\\
&= C_{\mathcal{F}}(J) - \frac{1 - \overline{f}_J^{FM}}{2}.
\end{aligned}
$$

Thus,

$$
condGD(J) = \frac{\overline{f}_J^{FM} - \overline{f}_J^{M}}{P(X_J \in \mathcal{F}, Y_J \in \mathcal{F})} = \frac{\overline{f}_J^{FM} - \overline{f}_J^{M}}{C_{\mathcal{F}}(J) - \frac{1-\overline{f}_J^{FM}}{2}}.
$$

The claim follows with $J = P_t$.

**Equation**

$$
C_{\mathcal{F}}(O_t(c)) = C_{\mathcal{F}}(O_t^N(c)) = c^T C_t \quad (2)
$$

**Proof:** We have

$$
\begin{aligned}
C_{\mathcal{F}}(O_t^N(c)) &= P(X_{O_t^N(c)} \in \mathcal{F})\\
&= \frac{1}{N} \sum_{i \in O_t^N(c)} P(X_i \in \mathcal{F})\\
&= \frac{1}{N} \sum_{i \in O_t^N(c)} P(X_i = X_{1i}) P(X_i \in \mathcal{F} | X_i = X_{1i}) + P(X_i = X_{2i}) P(X_i \in \mathcal{F} | X_i = X_{2i})\\
&= \frac{1}{N} \sum_{i \in O_t^N(c)} \frac{1}{2} \left( P(X_{1i} \in \mathcal{F} | X_i = X_{1i}) + P(X_{2i} \in \mathcal{F} | X_i = X_{2i}) \right)\\
&= \frac{1}{N} \sum_{i \in O_t^N(c)} \frac{1}{2} \left( P(X_{1i} \in \mathcal{F}) + P(X_{2i} \in \mathcal{F}) \right)\\
&= \frac{1}{2N} \sum_{i \in O_t^N(c)} P(X_{s_i} \in \mathcal{F}) + P(X_{d_i} \in \mathcal{F})\\
&= \frac{1}{2N} \sum_{a \in P_t} \#\{i \in O_t^N(c) : s_i = a \text{ or } d_i = a\} P(X_a \in \mathcal{F})\\
&= \sum_{a \in P_t} c_a P(X_a \in \mathcal{F})\\
&= c^T C_t
\end{aligned}
$$

**Equation**

$$condGD(O_t(c)) = \lim_{N\to\infty} condGD(O_t^N(c)) = \frac{c^T(f_{P_t}^{FM} - f_{P_t}^{M})c}{c^T C_t - \frac{1-c^T f_{P_t}^{FM} c}{2}} \tag{3}$$

**Proof:**

For brevity let $J_N = O_t^N(c)$. From the proof of Equation (1) we have:

$$condGD(J_N) = \frac{\overline{f}_{J_N}^{FM} - \overline{f}_{J_N}^{M}}{C_{\mathcal{F}}(J_N) - \frac{1-\overline{f}_{J_N}^{FM}}{2}}$$

In analogy to Wellmann and Pfeiffer (2009) it can be shown that

$$\overline{f}_{J_N}^{FM} = \frac{1}{(2N)^2}\left(\tilde{c}^T f_{P_t}^{FM}\tilde{c} + 2N - \tilde{c}^T \mathrm{Diag}(f_{P_t}^{FM})\right)$$
$$\overline{f}_{J_N}^{M} = \frac{1}{(2N)^2}\left(\tilde{c}^T f_{P_t}^{M}\tilde{c} + 2N - \tilde{c}^T \mathrm{Diag}(f_{P_t}^{M})\right),$$

where $\tilde{c} = 2Nc$, and $\mathrm{Diag}(X)$ is the vector that contains the diagonal elements of matrix $X$. It follows that

$$\lim_{N\to\infty} \overline{f}_{J_N}^{FM} = c^T f_{P_t}^{FM} c \quad \text{and} \quad \lim_{N\to\infty} \overline{f}_{J_N}^{M} = c^T f_{P_t}^{M} c. \tag{9}$$

Thus,

$$\lim_{N\to\infty} condGD(J_N) = \frac{c^T f_{P_t}^{FM} c - c^T f_{P_t}^{M} c}{c^T C_t - \frac{1-c^T f_{P_t}^{FM} c}{2}}.$$

**Equation**

$$\lim_{N\to\infty} \phi_B(O_t^N(c)) = c^T(f_{P_t}^{FM} - f_{P_t}^{M})c \tag{4}$$

**Proof:**

For brevity let $J_N = O_t^N(c)$. We have

$$\begin{aligned}
\lim_{N\to\infty} \phi_B(O_t^N(c)) &= \lim_{N\to\infty} P(X_{J_N} \neq Y_{J_N},\, X_{J_N} \in \mathcal{F},\, Y_{J_N} \in \mathcal{F}) \\
&= \lim_{N\to\infty} P(X_{J_N} \in \mathcal{F},\, Y_{J_N} \in \mathcal{F}) condGD(J_N) \\
&\overset{(8)}{=} \lim_{N\to\infty} \overline{f}_{J_N}^{FM} - \overline{f}_{J_N}^{M} \\
&\overset{(9)}{=} c^T(f_{P_t}^{FM} - f_{P_t}^{M})c
\end{aligned}$$

**Equation**

$$\lim_{N\to\infty} \phi_C(O_t^N(c)) \;=\; 1 - c^T f_{P_t}^M c \qquad (5)$$

**Proof:**

For brevity let $J_N = O_t^N(c)$. We have

$$\begin{aligned}
\lim_{N\to\infty} \phi_C(O_t^N(c)) &= \lim_{N\to\infty} P(X_{J_N} \neq Y_{J_N} \text{ and } (X_{J_N} \in \mathcal{F} \text{ or } Y_{J_N} \in \mathcal{F})) \\
&= \lim_{N\to\infty} P(X_{J_N} \neq Y_{J_N},\, X_{J_N} \in \mathcal{F},\, Y_{J_N} \in \mathcal{F}) \\
&+ 2\lim_{N\to\infty} P(X_{J_N} \neq Y_{J_N},\, X_{J_N} \in \mathcal{F},\, Y_{J_N} \in \mathcal{M}) \\
&= \lim_{N\to\infty} \phi_B(O_t^N(c)) \\
&+ 2\lim_{N\to\infty} P(X_{J_N} \in \mathcal{F},\, Y_{J_N} \in \mathcal{M}) P(X_{J_N} \neq Y_{J_N} | X_{J_N} \in \mathcal{F},\, Y_{J_N} \in \mathcal{M}) \\
&= \lim_{N\to\infty} \phi_B(O_t^N(c)) + \lim_{N\to\infty} 2P(X_{J_N} \in \mathcal{F},\, Y_{J_N} \in \mathcal{M}) \\
&\overset{(4),(7)}{=} c^T(f_{P_t}^{FM} - f_{P_t}^M)c + \lim_{N\to\infty}(1 - \overline{f}_J^{FM}) \\
&\overset{(9)}{=} 1 - c^T f_{P_t}^M c
\end{aligned}$$

**Equation**

$$\lim_{N\to\infty} \phi_D(O_t^N(c)) \;=\; \frac{c^T(f_{P_t}^{FM} - f_{P_t}^M)c}{c^T Q_t c} \qquad (6)$$

**Proof:**

From Equation 3 we have

$$\begin{aligned}
\lim_{N\to\infty} condGD(J_N) &= \frac{c^T f_{P_t}^{FM} c - c^T f_{P_t}^M c}{c^T C_t - \frac{1 - c^T f_{P_t}^{FM} c}{2}} \\
&= \frac{c^T(f_{P_t}^{FM} - f_{P_t}^M)c}{\frac{c^T C_t \mathbf{1}^T c + c^T \mathbf{1} C_t^T c}{2} - \frac{c^T \mathbf{1}\mathbf{1}^T c - c^T f_{P_t}^{FM} c}{2}} \\
&= \frac{c^T(f_{P_t}^{FM} - f_{P_t}^M)c}{c^T \frac{1}{2}\left(C_t \mathbf{1}^T + \mathbf{1} C_t^T - \mathbf{1}\mathbf{1}^T + f_{P_t}^{FM}\right) c}
\end{aligned}$$
